# Characterization of *Brassica napus* L. genotypes utilizing sequence-related amplified polymorphism and genotyping by sequencing in association with cluster analysis

**DOI:** 10.1007/s11032-016-0576-6

**Published:** 2016-11-10

**Authors:** Corey J. Lees, Genyi Li, Robert W. Duncan

**Affiliations:** Faculty of Agricultural and Food Sciences, Department of Plant Science, University of Manitoba, 222 Agriculture Building, Winnipeg, MB R3T 2N2 Canada

**Keywords:** GBS, SRAP, Genetic distance, Cluster analysis, Heterosis, *Brassica napus*

## Abstract

Identifying parental combinations that exhibit high heterosis is a constant target for commercial *Brassica napus* L. hybrid development programs. Finding high heterotic parental combinations can require hundreds of test crosses and years of yield evaluation. Heterotic pool development could be used to divide breeding material into specific breeding pools and focus the number of parental combinations created. Here, we report the genotypic characterization of 79 *B. napus* genotypes by calculating genetic distance based on sequence-related amplified polymorphism (SRAP) and genotyping by sequencing (GBS) in association with a neighbour-joining clustering algorithm. Despite the different genotypic analyses, neighbour-joining cluster analysis based on genetic distance of SRAP and GBS produced similar clusters. Homology between SRAP and GBS clusters was approximately 77 % when manually comparing clusters and 68 % when comparing clusters using Compare2Trees. This research demonstrates that SRAP can have similar efficacy when compared to next-generation sequencing technology for heterotic pool classification. This information may provide an important breeding scaffold for the development of hybrid cultivars based upon genetic distance and cluster analysis.

**Electronic supplementary material:**

The online version of this article (doi:10.1007/s11032-016-0576-6) contains supplementary material, which is available to authorized users.

## Introduction

*Brassica napus* L. (AACC *n* = 19) has been heralded as one of the most important global oilseed crops, second only to soybean (*Glycine max* L.) for total annual production (Carré and Pouzet 2014; Wittkop et al. 2009; Lin et al. 2013). This large-scale production is attributed to the improvement of canola quality (<2 % erucic acid, <30 μmol/g glucosinolates) and specialty canola cultivars that produce high stability oil for human consumption and meal for animal feed (Rahman 2013). Additionally, high erucic acid rapeseed (HEAR) cultivars also contribute valuable industrial oil for lubricants and slip agents for niche oleochemical markets (Wittkop et al. 2009; McVetty and Duncan 2014). Hybrid *B. napus* cultivars have replaced their open-pollinated counterparts due to the exploitation of heterosis (Rahman 2013). Heterosis occurs when progeny outperform both parents in a variety of agronomic traits but specifically seed yield (Shull 1908; Radoev et al. 2008). Therefore, hybrid cultivars that possess increased yield and superior uniformity in the F_1_ generation are highly sought after by producers to continually match global demand (Riaz and Quiros 2011). Finding parental combinations that exhibit high heterotic gains may require hundreds of test crosses and years of yield evaluation. To alleviate this problem, heterotic pool development has been suggested as a method to separate germplasm and breeding genotypes into distinct clusters for breeding purposes (Rahman 2013; Odong et al. 2011; Girke et al. 2012).

Distinguishing genotypes and the development of heterotic pools can be based on genetic distance and cluster analysis (Ali et al. 1995; Riaz and Quiros 2011; Jesske et al. 2013). Genetic distance is a measure of genetic divergence between species or between individuals and populations within the same species (Nei 1987). Genetic distance can be calculated using a variety of genotypic data sets produced using multiple molecular characterization methods (Jain et al. 1994; Becker et al. 1995; Lombard et al. 2000) along with numerous mathematical formulas (Nei 1972; Cavalli-Sforza and Edwards 1967; Reynolds et al. 1983; Tamura and Nei 1993). Several forms of cluster analysis exist including Ward’s method (Ward 1963), unweighted pair group method with arithmetic mean [UPGMA (Sokal and Michener 1958)] and neighbour-joining [NJ (Saitou and Nei 1987)] which are all agglomerative (bottom-up) clustering techniques. Applying clustering algorithms to genetic distance models allows for the grouping of genotypes with a short genetic distance together into a heterotic cluster or pool (Odong et al. 2011). Genetic distance and cluster analysis have previously been employed in *B. napus* hybrid development (Diers et al. 1996; Riaz et al. 2001; Yu et al. 2005). Previous research has shown that *B. napus* parental combinations from different pools or clusters exhibit higher heterosis than those using parents from the same cluster (Ali et al. 1995; Riaz and Quiros 2011). Although clustering techniques have been used for decades in plant breeding, there is no scientific consensus on which clustering method produces the most accurate cluster set for breeding purposes (Mouchet et al. 2008; Odong et al. 2011). Therefore, the molecular characterization method, genetic distance method and clustering method create a large combination of experimental designs that can be used to develop and explore heterotic pools for hybrid development.

Sequence-related amplified polymorphism (SRAP, Li and Quiros 2001) has been a popular molecular method for the identification of genetic diversity in many crop species including *Cucurbita pepo* L. (Ferriol et al. 2003), *Prunus persica* L. (Ahmad et al. 2004) and *Lycopersicon esculentum* L. (Ruiz et al. 2005). In addition, SRAP has previously been successful in associating genetic distance calculated in the presence/absence of markers with hybrid heterosis based on cluster analysis in *B. napus* (Riaz et al. 2001; Riaz and Quiros 2011). However, today’s next-generation sequencing technologies have reduced the cost of DNA sequencing, allowing the ability to evaluate genetic diversity based on single nucleotide polymorphisms (SNPs) (Nielsen et al. 2011; Elshire et al. 2011). Genotyping by sequencing (GBS) is one such next-generation method that can be used for SNP discovery which uses reduced genome representation with methylation-sensitive restriction enzymes (Elshire et al. 2011). Through methylation sensitivity, repetitive regions of the genome can be avoided which simplifies the computational challenge of genome alignment with large, polyploidy genomes (Elshire et al. 2011). To date, several important crop species including corn (*Zea mays* L.), barley (*Hordeum vulgare* L.) and rice (*Oryza sativa* L.) have used GBS for a multitude of downstream applications (Elshire et al. 2011; Liu et al. 2014; Spindel et al. 2013).

The objective of this study was to calculate genetic distance between 79 *B. napus* genotypes using Nei’s standard genetic distance based on SRAP presence/absence genotyping and the Tamuri–Nei genetic distance formula based on SNPs discovered through GBS. A neighbour-joining clustering method was then used on each separate genetic distance method, and the results were compared. Ultimately, the goal of this research is to improve heterotic pool definitions through multiple techniques and to find a system that will reduce the overall time and space required for discovering high heterotic parental combinations.

## Materials and methods

### *Brassica napus* genotypes

Seventy-nine *B. napus* genotypes were selected for this study (Table 1). Most genotypes are considered spring habit germplasm developed for western Canada, with the addition of several European and resynthesized *B. napus* genotypes. Several genotypes are of canola quality, while the vast majority are considered as HEAR. Of the 79 genotypes, 38 are *ogu*-INRA restorers and 41 are open-pollinated genotypes (maintainers or B-lines in the *ogu*-INRA pollination control system).

**Table 1:** Seventy-nine spring habit *Brassica napus* L. genotypes with origin, quality and maintainer or restorer designation for the *ogu*-INRA pollination control system

Identification	Origin	Quality	Maintainer/restorer
NEW-620-R	U of M	HEAR	Restorer
NEW-621-R	U of M	HEAR	Restorer
Castor-R	U of M	HEAR	Restorer
UMI-71-R	European	HEAR	Restorer
UMS-189-R	European	HEAR	Restorer
12DH378-R	U of M	HEAR	Restorer
12DH377-R	U of M	HEAR	Restorer
RRHR503-R	U of M	HEAR	Restorer
RRHR204-R	U of M	HEAR	Restorer
RRHR404-R	U of M	HEAR	Restorer
RRHR5815-R	U of M	HEAR	Restorer
RRHR5819-R	U of M	HEAR	Restorer
08C702-R	U of M	HEAR	Restorer
08C712-R	U of M	HEAR	Restorer
08C847-R	U of M	HEAR	Restorer
Red River 1997-R	U of M	HEAR	Restorer
Red River 1826-R	U of M	HEAR	Restorer
Red River 1852-R	U of M	HEAR	Restorer
Red River 1997-R2	U of M	HEAR	Restorer
UMI-55-R	European	HEAR	Restorer
UMI99-R	European	HEAR	Restorer
12DH384-R	U of M	HEAR	Restorer
12DH430-R	U of M	HEAR	Restorer
12DH478-R	U of M	HEAR	Restorer
12DH915-R	U of M	HEAR	Restorer
12DH949-R	U of M	HEAR	Restorer
11DH91-R	U of M	HEAR	Restorer
11DH92-R	U of M	HEAR	Restorer
11DH97-R	U of M	HEAR	Restorer
11DH108-R	U of M	HEAR	Restorer
11DH109-R	U of M	HEAR	Restorer
11DH114-R	U of M	HEAR	Restorer
11DH122-R	U of M	HEAR	Restorer
11DH137-R	U of M	HEAR	Restorer
11DH144-R	U of M	HEAR	Restorer
11DH148-R	U of M	HEAR	Restorer
11DH149-R	U of M	HEAR	Restorer
11DH162-R	U of M	HEAR	Restorer
Red River 1826-B	U of M	HEAR	Maintainer
Red River 1852-B	U of M	HEAR	Maintainer
Red River 1997-B	U of M	HEAR	Maintainer
Red River 1861-B	U of M	HEAR	Maintainer
RRHR503-B	U of M	HEAR	Maintainer
RRHR204-B	U of M	HEAR	Maintainer
RRHR404-B	U of M	HEAR	Maintainer
RRHR5815-B	U of M	HEAR	Maintainer
RRHR5819-B	U of M	HEAR	Maintainer
08C702-B	U of M	HEAR	Maintainer
08C712-B	U of M	HEAR	Maintainer
08C847-B	U of M	HEAR	Maintainer
RRHR9707-B	U of M	HEAR	Maintainer
UMI-B	European	HEAR	Maintainer
UMSA-B	European	HEAR	Maintainer
UMSH-B	European	HEAR	Maintainer
Venus-B	U of M	HEAR	Maintainer
Neptune-B	U of M	HEAR	Maintainer
Castor-B	U of M	HEAR	Maintainer
Mill 03-B	U of M	HEAR	Maintainer
Mercury-B	U of M	HEAR	Maintainer
Hero-B	U of M	HEAR	Maintainer
08C344-B	U of M	HEAR	Maintainer
Reston-B	U of M	HEAR	Maintainer
79R713-B	U of M	HEAR	Maintainer
79R714-B	U of M	HEAR	Maintainer
79R728-B	U of M	HEAR	Maintainer
79R729-B	U of M	HEAR	Maintainer
ER2-B	Resynthesized	HEAR	Maintainer
ER3-B	Resynthesized	HEAR	Maintainer
ER7-B	Resynthesized	HEAR	Maintainer
ER22-B	Resynthesized	HEAR	Maintainer
ZSDH2602-B	Resynthesized	HEAR	Maintainer
Topas-B	U of M	Canola	Maintainer
Polo-B	U of M	Canola	Maintainer
Sentry-B	U of M	Canola	Maintainer
Global-B	U of M	Canola	Maintainer
Westar-B	U of M	Canola	Maintainer
Apollo-B	U of M	Low linolenic	Maintainer
Stellar-B	U of M	Low linolenic	Maintainer
02R276-B	U of M	High oleic low linolenic	Maintainer

*U of M* University of Manitoba, *HEAR* high erucic acid rapeseed

### Greenhouse production

Initially, three replicates for each genotype were planted at a depth of 1 cm into plastic 4 × 3-well cell packs (13 × 13 × 5 cm) containing Sunshine Metro Mix potting soil (Sungro® Horticulture, MA, USA) from March 5–12, 2014. Plants were grown in a growth chamber (temperature day 22 °C, night 18 °C; light cycle 16 h light, 8 h dark) and watered daily. At the two-leaf stage [14 days after planting (DAP)], each plant was transferred to a plastic grower pot (14.5 × 15 cm) using Sunshine Metro Mix potting soil. Pots were transferred to an Argus-controlled greenhouse (Argus Control Systems Ltd., Surrey, BC, Canada) with the following specifications (temperature high 25 °C, low 22 °C; relative humidity 40–50 %; light cycle 16 h light, 8 h dark) and watered daily. Fertilizer was applied twice, once at the time of transplanting (14 DAP) and once during flowering (50 DAP) using Plant-Prod® water soluble fertilizer (10-52-10) at a concentration of 15 g/3.78 l. Insect populations were controlled with Intercept™ 60 WP (imidacloprid, Bayer Environmental Science, Research Triangle Park, NC, USA) added several days after transplant (20 DAP) with a concentration of 4.1 g/1000 seedlings with 15 l of solution per 100 m^2^ of seeding trays. All mixing and application procedures were followed as per the manufacturer’s instructions.

### DNA extraction

For the SRAP genotyping method, DNA was extracted from fresh leaves in April of 2014 by the cetyltrimethylammonium bromide (CTAB) method (Doyle and Doyle 1990) with minor modifications. A 500–600 mg leaf sample was crushed using a mortar and pestle with liquid nitrogen. The ground leaf tissue was added to a 15-ml centrifuge tube. Six milliliters of preheated extraction buffer (500 ml of 2 % CTAB, 100 mM Tris–HCl, pH 8.0, 1.4 M NaCl, 20 mM EDTA) was then added, and the tube and was vortexed and incubated for 90 min in a 65 °C water bath. The tube was cooled to room temperature, and 7 ml of chloroform–isoamyl alcohol (24:1) was added. The solution was mixed gently for 10 min and centrifuged at 4600 RPM for 16 min. The supernatant was transferred to a new 15-ml tube; 0.5 volumes of 2-propanol were added and mixed gently to precipitate the DNA. The mixture was centrifuged at 4600 RPM for 5 min; the supernatant was removed, and the DNA pellet was washed with 8 ml 70 % (*v*/*v*) ethanol. The pellet was then air-dried and resuspended in 3 ml of double-distilled water.

Due to purity requirements for GBS, a Qiagen® DNeasy Plant Mini Kit (Qiagen, Valencia, CA, USA) was utilized to ensure ultra-pure DNA. All procedures were followed as per manufacturer’s instructions for fresh tissue DNA extraction and purification with the addition of 10-min total elusion time (2 × 5 min).

### DNA concentration

DNA quantity for SRAP was determined using a Thermo Scientific NanoDrop 2000 spectrophotometer in conjunction with software NanoDrop 2000 (Thermo Fisher Scientific Inc., MA, USA) with all protocols followed as per manufacturer’s instructions for double-stranded DNA quantification. All samples achieved a minimum DNA concentration of 30 ng/μl with a 260/280 ratio of ≥1.8. All DNA concentrations for GBS were quantified using a Life Technologies Qubit® Fluorometer (Life Technologies, Carlsbad, CA, USA) with all protocols followed as per manufacturer’s instructions for double-stranded DNA quantification. All samples reached a minimum concentration of 50 ng/μl as per GBS requirements (http://www.biotech.cornell.edu/brc/genomic-diversity-facility).

### Sequence-related amplified polymorphism

Sequence-related amplified polymorphism is a polymerase chain reaction (PCR) method, which is designed to amplify open reading frames (ORFs) using a variable forward and reverse primer system (Li and Quiros 2001). Each primer is 17–18 base pairs long with the forward primer containing a core sequence of CCGG. This forward core sequence targets ORFs due to the known distribution that exons are GC rich (Li and Quiros 2001). The reverse primer has a core sequence of AATT near the 3′ region to target introns and promoter regions that are typically AT rich (Li and Quiros 2001). Together, these primer combinations create polymorphic DNA bands that are separated through electrophoresis in polyacrylamide gels and visualized through autoradiography (Li and Quiros 2001). The presence/absence scoring is then applied to the visualized polymorphic bands, and genotypes can be separated based on scoring (Li and Quiros 2001).

Primer design followed the protocol of Li and Quiros (2001). Supplemental Table 1 displays the base pair sequence of all primers used in this research. Primer combinations consisted of 29 forward and reverse primer sets used to genotype 79 *B. napus* genotypes (Supplemental Table 2).

### Polymerase chain reaction

Ten microlitre aliquots of PCR master mix were allocated into a 384-well plate. The master mix was composed of 8.6 μl ddH_2_O, 1 μl 10× PCR buffer (500 mM KCl, 100 mM Tris, 1 % Triton, 1.5 mM MgCl_2_, pH 9.3), 0.15 μl dNTPs, 0.15 μl forward primer (Table 2), 0.15 μl reverse primer (Table 2) and 0.15 μl *Taq* polymerase. DNA was transferred via a stainless steel 96 spike stamping plate and sealed with a PCR plate cover for the PCR procedure. PCR was completed using the following parameters: temperature cycle initiated at 94 °C for 3 min with cycle 2 at 94 °C for 55 s, cycle 3 was 35 °C for 55 s and cycle 4 was 72 °C for 55 s. Cycles 2–4 were repeated five times. Cycle 5 was set to 94 °C for 55 s; cycle 6 was 50 °C for 55 s, and cycle 7 was set to 72 °C for 55 s. Cycles 5–7 were repeated 30 times. After the final cycle was completed, the PCR products were separated by denaturing acrylamide gels and detected by autoradiography with an ABI Prism 3130XL in association with GenScan® software (V.3.7) (Applied Biosystems, Life Technologies, Carlsbad, CA, USA) (Li and Quiros 2001).

**Table 2:** Total number of sequence reads, good barcoded reads and resulting tags for each sequence run of genotyping by sequencing on 95 *Brassica napus* genotypes

	Barcodes found in lane	Total number of reads per lane^a^	Total number of good barcoded reads^b^	Resulting number of tags^c^
Run 1	96	132,278,340	126,177,590	8,110,178
Run 2	96	121,989,805	116,807,543	6,580,155

^a^A read is a single sequence generated by the GBS assay

^b^A good barcoded read was recorded if (1) the read perfectly matched the barcode sequence and the four base remnant *Ape*K1 cut site, (2) there were no adapter/adapter dimers and (3) contained no *N*s (missing) up to the trim length (Glaubitz et al. 2014)

^c^A tag is a unique sequence from the good barcoded reads

### Genotyping by sequencing

GBS is a method for measuring SNPs and utilizes the Illumina® next-generation sequencing technology (Elshire et al. 2011). GBS is a highly multiplexed PCR method that uses a reduced representation of genome complexity through the use of restriction enzymes (REs) that are methylation sensitive (Elshire et al. 2011). This greatly simplifies sequencing and alignment procedures allowing for deep coverage in gene-rich regions (Chen et al. 2013; Elshire et al. 2011). Following RE digestion, adapter barcodes are ligated to the RE cut site allowing many samples to be pooled into one Illumina flow cell greatly reducing cost (Elshire et al. 2011; Chen et al. 2013). Generally, millions of sequence tags (64 bp reads) are generated and 10,000s to 100,000s of SNPs can be called with a very high degree of accuracy through a novel GBS computational pipeline, Tassel (Bradbury et al. 2007; Elshire et al. 2011; Glaubitz et al. 2014).

One 96-well plate (Eppendorf twintec PCR plate 96 well) (caps: Thermo Scientific PCR 8 Strip Flat Caps) with DNA samples of 95 *B. napus* genotypes was submitted to Cornell University Institute of Biotechnology (http://www.biotech.cornell.edu/brc/genomic-diversity-facility) where GBS was completed as per the protocol defined by Elshire et al. (2011). *Ape*KI (GCWGC, where W is A or T) was the restriction enzyme chosen at a 95-plex level. All bioinformatics analysis (SNP calling) was completed by Cornell University Institute of Biotechnology using the Tassel computation pipeline V. 3.0.166 (Bradbury et al. 2007; Glaubitz et al. 2014) and the *B. napus* reference genome (Chalhoub et al. 2014). Genome alignment was generated with Burrows–Wheeler transform algorithm (BWA) version 0.7.8-r455 (Li and Durbin 2010; Li and Homer 2010).

### Cluster analysis

For SRAP, each presence/absence marker was scored using a binary system of 1 for present and 0 for absent creating a matrix. Genetic distance using Nei’s standard genetic distance (Nei 1972) formula was calculated using the software Powermarker (V3.25) (Liu and Muse 2005; http://statgen.ncsu.edu/powermarker/) based upon the binary matrix. This calculation created a new matrix of 79 × 79 genotypes with the genetic distance between each genotype displayed. Neighbour-joining cluster analysis (Saitou and Nei 1987) was then applied to the genetic distance matrix using the software Powermarker (V3.25). This created a reference tree with branch lengths between genotypes approximately equal to the genetic distance between genotypes. Tree robustness was tested with the generation of 1000 bootstrapping replicates (Felsenstein 1985). The reference tree file and the 1000 bootstrapping replicate trees were exported as Newick format into Mega 6 (V.6.06 [6140226] Tamura et al. 2013; http://www.megasoftware.net/) and exported as Newick file to be compatible with the software Geneious V.8.05 (Kearse et al. 2012; http://www.geneious.com/download). Consensus tree construction based on the 1000 bootstrapping replicates was completed in Geneious V.8.05 with the following parameters: support threshold set to 0, topology threshold set to 0, burn in set to 0 and a greedy clustering model.

For GBS, 80,005 filtered biallelic SNPs were imported into the software program Geneious V.8.05. Only 79 of the 95 genotypes were analysed to match the SRAP analysis. Genetic distance was calculated by Geneious based on the Tamura–Nei distance model (Tamura and Nei 1993), and the subsequent distance matrix was clustered using neighbour-joining method (Saitou and Nei 1987). This created a reference tree with branch lengths between genotypes approximately equal to the genetic distance between genotypes. Tree robustness was tested with 1000 bootstrapping replicates in Geneious V.8.05 with the following parameters: support threshold set to 0, topology threshold set to 0, burn in set to 0 and a greedy clustering model.

### Cluster similarity

Cluster similarity was explored on a cluster-by-cluster level, where each conserved cluster (*K*) was compared to each other for *n* number of matches, and each match for each cluster was signified as a match percentage for each cluster. This consisted of a branch-to-branch comparison of conserved clusters within each tree. This allows a computation of the similarity of each conserved cluster and the overall similarity between dendrograms using a match percent despite changes in topology. Secondly, cluster similarity was computed using the Java applet Compare2Trees (Nye et al. 2006; http://www.mrc-bsu.cam.ac.uk/personal/thomas/phylo_comparison/comparison_page.html.). In short, two phylogenetic trees, *T*_1_ and *T*_2_, that share the same set of leaves (*L*) can be compared and can be either rooted or unrooted. The comparison algorithm has two stages: First, every pair of edges (*i*, *j*) with *i* ∈ *T*_1_ and *j* ∈ *T*_2_ is assigned a score *s* (*i*, *j*) that reflects the topological similarity of the branches *i* and *j*. Secondly, branches in the two trees are paired up to optimize the overall score creating a branch-to-branch comparison (Nye et al. 2006).

## Results

### Sequence-related amplified polymorphism

Twenty-nine forward and reverse primer combinations amplified through PCR and visualized through autoradiography resulted in 293 polymorphic bands between the 79 *B. napus* genotypes. On average, each primer combination amplified 10 polymorphic bands per genotype (Supplementary Fig. 1).

### Genotyping by sequencing

The first GBS run produced ∼126 million barcode reads, 84 % of the minimum 150 million barcode reads that Cornell’s Institute of Biotechnology has set as their standard. As a result, our GBS material was sequenced a second time generating an additional ∼116 million barcode reads and combined with the first sequencing run (Table 2). This combined pool generated ∼8,110,000 and ∼6,580,000 unique sequence tags, respectively, for a combined total of 1,631,637 filtered sequence tags. Filtered sequence tags are tags at or above a defined threshold for all taxa (samples) in the experiment and were used for genome alignment (Glaubitz et al. 2014). Of those filtered sequence tags, 925,657 (56.7 %) aligned to unique positions, 420,244 (25.8 %) aligned to multiple positions and 285,736 (17.5 %) could not be aligned to the reference genome. From the alignment results, all unique aligned filtered sequence tags (925,657 or 56.7 %) were used for SNP calling based on the reference genome provided (Chalhoub et al. 2014). The resulting SNPs called from the unique aligned positions were divided into three distinct categories [VCF, HapMap (unfiltered), HapMap (filtered)]. The VCF SNPs and HapMap SNPs were called independently, and variation between the two formats was expected. VCF SNPs were called using the algorithm from Catchen et al. (2011) called Stacks, whereas HapMap SNPs were called in Tassel (Bradbury et al. 2007; Glaubitz et al. 2014). Stacks SNP calling resulted in 382,560 VCF SNPs. Tassel SNP calling generated 179,974 unfiltered SNPs stored in HapMap format. HapMap SNPs were filtered on missingness and allele frequency which generated 80,005 high-quality bi-allelic SNPs.

### Cluster analysis

**Fig. 1:**
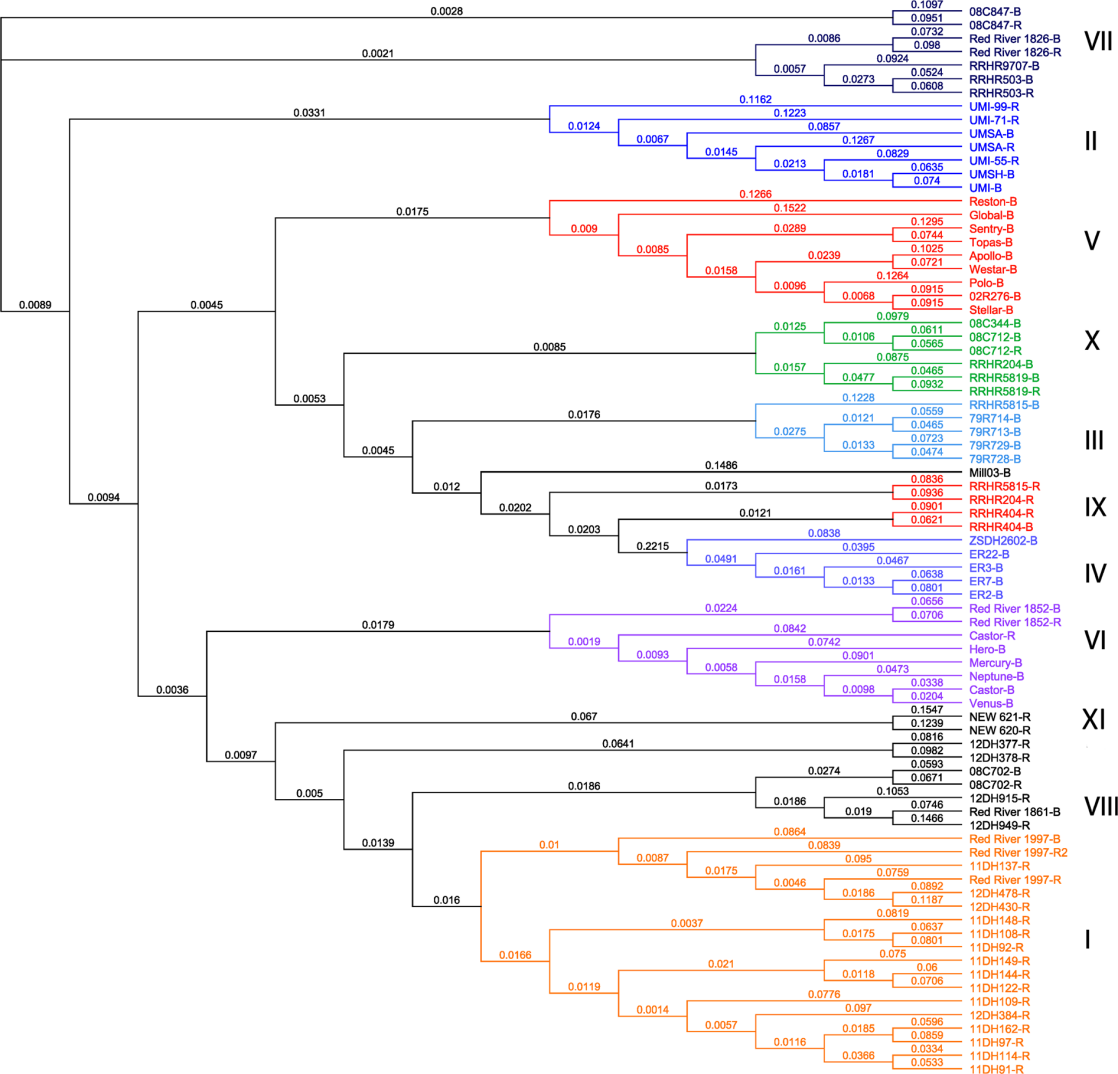
Neighbour-joining dendrogram clustered using Nei’s standard genetic distance based on 293 SRAP polymorphic bands obtained through sequence-related amplified polymorphisms on 79 *Brassica napus* genotypes visualized in Geneious V.8.05. Distinct clusters have been numbered and colour-coded for ease of viewing. Each genotype is either a maintainer (-B) or restorer (-R) in the *ogu*-INRA pollination control system

**Fig. 2:**
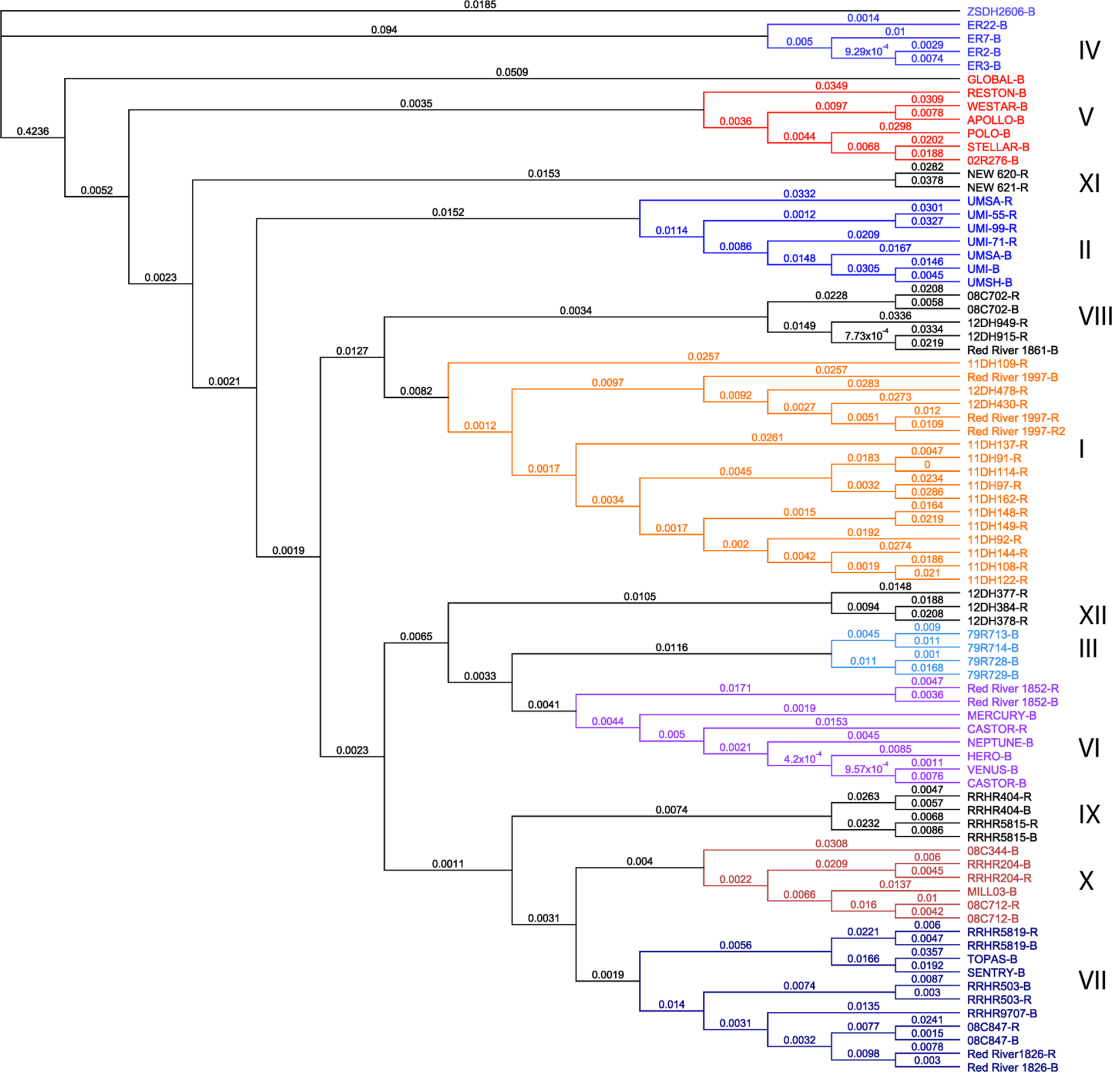
Neighbour-joining dendogram based on Tamura–Nei’s genetic distance calculated on 80,005 SNPs obtained from genotyping by sequencing on 79 *Brassica napus* genotypes visualized in Geneious (V.8.05). Distinct clusters have been numbered and colour-coded for ease of viewing. Each genotype is either a maintainer (-B) or restorer (-R) in the *ogu*-INRA pollination control system

The neighbour-joining cluster analysis based on the SRAP genetic distance matrix produced a reference dendrogram with 11 distinct heterotic clusters (Fig. 1). Dendrogram robustness was tested through 1000 bootstrapping replications (Supplementary Fig. 2). The bootstrapping dendrogram also produced 11 heterotic clusters. However, only five clusters (II, IV, V, VII and IX) remained identical over 1000 replications. These can be considered high confidence heterotic clusters, although only minor genotype movement was observed throughout the other non-identical clusters. The neighbour-joining cluster analysis based on the GBS distance matrix produced a reference dendrogram with 12 heterotic clusters (Fig. 2). Again, tree robustness was tested with 1000 bootstrapping replications (Supplementary Fig. 3). Between the GBS reference tree and the bootstrapping tree (Fig. 2 and Supplemental Fig. 3), only two heterotic clusters (IV and V) did not remain identical over 1000 replications. The GBS bootstrapping tree showed remarkable robustness as many nodes have a 90 % or higher clustering percent over 1000 bootstrapping replications (Felsenstein 1985), and considerable confidence can be given to a tree that is supported by >80 % of bootstrapping replicates (Zharkikh and Li 1992). On the other hand, little confidence can be given to a tree that is supported by <75 % of the replicates (Zharkikh and Li 1992). This may apply to the SRAP bootstrapping tree as it seemed to vary over 1000 replications, and its node length ranged from approximately 1.3 to 99.5 % intervals. Comparing all dendrograms, cluster II remained identical through the different methods and replicates. Cluster II is represented by genotypes of European descent.

### Genetic distance

SRAP genetic distance based on Nei’s genetic distance formula calculated on 293 polymorphic bands had a genetic distance range of 0.08 between genotypes 11DH91-R and 11DH114-R to 0.74 between genotypes ER2-B and NEW-620-R (Fig. 1). GBS genetic distance was calculated using the Tamura–Nei formula using 80,005 SNPs, which had a range of 0.0047 between genotypes 11DH91-R and 11DH114-R to 0.629 between genotypes ER3-B and 08C847-R (Fig. 2).

### Cluster similarity

Cluster similarity was investigated between the two genotypic methods of SRAP and GBS in association with Nei’s standard genetic distance and the Tamura–Nei distance model, respectively. Cluster topology differed between the genetic distance calculated on Nei’s standard genetic distance based on SRAP and the genetic distance calculated on the Tamura–Nei model based on GBS despite using the same neighbour-joining algorithm (Table 3). However, distinct clusters contained similar genotypes between the two methods. Clusters II, IV, VI and XI are all examples where all genotypes remained identical over both methods.

**Table 3:** Manual comparison of similarity for each conserved cluster between the genetic distances of sequence-related amplified polymorphism and genotyping-by-sequencing dendrograms for individual genotypes (*n*)

Conserved clusters between dendrograms	SRAP (*n*)^a^	GBS (*n*)^b^	Match %^c^
I	18	17	94.4
II	7	7	100
III	5	4	80
IV	5	5	100
V	9	7	78
VI	8	8	100
VII	7	11	64
VIII	7	5	71
IX	5	4	60
X	6	6	71
XI	2	2	100
XII	0	3	0
Total (*n*) and mean match percent (%)	79	79	76.5

^a^The total number of genotypes separated by neighbour-joining cluster analysis based on SRAP genetic distance

^b^The total number of genotypes separated by neighbour-joining cluster analysis based on GBS genetic distance

^c^Match percent obtained by dividing the smaller number of genotypes per cluster by the larger number of genotypes per cluster

Despite the differences in cluster placement, each cluster between the two dendrograms shows highly conserved clusters for specific genotypes (Figs. 1 and 2). There is an approximate homology of 77 % between all genotypes in all clusters when manually compared. The Java applet Compare2Trees was implemented for a branch-to-branch computational comparison (Nye et al. 2006). Compare2Trees found an approximate homology of 68 % between the SRAP and GBS trees.

## Discussion

Several primer combinations for SRAP have been previously been reported and found to be successful for differentiating *B. napus* genotypes (Li and Quiros 2001; Sun et al. 2007; Wen et al. 2006; Riaz et al. 2001). Sun et al. (2007) constructed an ultra-dense genetic map using 1634 SRAP primer combinations to produce 13,551 mapped markers. Wen et al. (2006) discovered 123 polymorphic fragments using 25 SRAP primer combinations, and Riaz et al. (2001) found 118 polymorphic bands based on 18 forward and reverse SRAP primer combinations. Here, we report 293 polymorphic bands with 29 forward and reverse primer combinations for 79 *B. napus* genotypes.

Genotyping by sequencing is a relatively new protocol for high-throughput SNP detection (Elshire et al. 2011). There is currently little GBS data published for *B. napus* diversity. We report that 285,736 tags or 17.5 % of filtered tags could not be aligned to the *B. napus* reference genome (Chalhoub et al. 2014). Elshire et al. (2011) reported that only 2 % of parental maize line B73 filtered tags could not be aligned to the maize reference genome (B73 RefGen V.1). Elshire et al. (2011) found that this 2 % of non-aligning reads were not present in the reference genome. In the current research, 17.5 % could not be aligned, and in conjunction with the Elshire et al. (2011) findings, these sequencing tags are probably not contained within the reference genome. Currently, the *B. napus* genome is of winter habit and is an open-pollinated genotype (Chalhoub et al. 2014). This may explain a vast majority of non-aligning reads as our material is considered to be spring habit and 38 of the 79 genotypes contain restorer introgressions from *Raphanus sativa* L. for use in the *ogu*-INRA pollination control system (Heyn 1976; Delourme and Eber 1992; Gourret et al. 1992; Delourme et al. 1994). These two differences may contribute to the unaligned sequences; however, this hypothesis warrants further investigation.

Genetic distance has been a well-cited mathematical tool for the determination of species and/or individual relatedness (Ali et al. 1995; Riaz et al. 2001; Yu et al. 2005; Jesske et al. 2013). However, very few studies present multiple genetic distance methods with the same population or genotypes with the same clustering method for the purpose of evaluating genetic distance measures. Here, the ultimate goal is to investigate which genetic distance method can produce highly accurate heterotic pools for the purpose of predicting heterosis. To pursue this endeavour, intra-cluster and inter-cluster hybrids from the current dendrograms need to be evaluated to gauge which genetic distance method has greater predictive power. However, despite these different methods, genotypes 11DH91-R and 11DH114-R produced the smallest genetic distance using both methods. In addition, the largest values obtained for both genetic distance methods involved Canadian *B. napus* genotypes compared to resynthesized *B. napus*, and this is in agreement with Jesske et al. (2013) who presented evidence that resynthesized *B. napus* genotypes contain genetic diversity not seen in elite breeding lines. This experimental evidence lends credibility to both SRAP and GBS methods as each separate mathematical formula calculated the shortest genetic distance between the same pair of genotypes and also produced the largest genetic distance between Canadian *B. napus* genotypes and resynthesized genotypes.

The comparison between the genetic distance dendrograms derived from SRAP and GBS (Figs. 1 and 2) was remarkably similar despite using different genetic distance formulas and different genotypic methods (77 % homology based on manual match percent and 68 % homology calculated by the Java applet Compare2Trees). The close association in percentage shows that these trees are similar; however, when bootstrapping values were incorporated, the GBS bootstrapping tree was stronger and more robust as opposed to the SRAP bootstrapping dendrogram (Supplemental Figs. 2 and 3). Unfortunately, the program Compare2Trees cannot compare bootstrapping trees (Nye et al. 2006). However, across all dendrograms, a visual inspection shows that cluster II remained identical. This supports the conclusions of Diers et al. (1996) and Cuthbert et al. (2009). Specifically, Cuthbert et al. (2009) showed that European-derived breeding material was distinct from Canadian high erucic acid rapeseed material based on heterotic performance and cluster II in this analysis retains only European-derived genotypes.

From a *B. napus* breeding standpoint, it is well cited that inter-cluster hybrids exhibit higher heterosis than intra-cluster hybrids (Grant and Beversdorf 1985; Riaz et al. 2001; Riaz and Qiuros 2011). This assumption is also well documented in maize hybrid breeding as many commercial hybrids are from complimentary heterotic pools (e.g. Reid Yellow Dent and Lancaster Sure Crop) (Lu et al. 2009; Chen et al. 2015). The conserved clusters between SRAP and GBS (II, IV, VI and XI) suggest that the genotypes within each cluster may be more important for heterotic gains than cluster placement in the overall topology of the dendrogram, since topologies shift between methods. Genotypic placement within clusters can be considered highly accurate given origin and pedigree information. For instance, cluster I for both methods contains mostly double haploid (DH) material and Red River 1997, a parental genotype for most of the DH material. Cluster II was all European-sourced material; cluster IV was all resynthesized genotypes, and cluster VI contains genotypes released by the University of Manitoba (Scarth et al. 1991; Scarth et al. 1995; McVetty et al. 1996a,b; McVetty et al. 1998; McVetty et al. 2006). Further investigation between inter-cluster and intra-cluster hybrids as well as cluster placement and the genetic distance between each parental genotype would prove extremely useful for developing a breeding schematic based on genetic distance and cluster analysis for *B. napus* hybrids. Since cluster II was conserved across all methods and replicates, this European-derived cluster is distinct and future inter-cluster hybrids should be explored using this cluster.

From a monetary standpoint, GBS was outsourced to Cornell University Institute of Biotechnology which (as of 2014) roughly had a price tag of US$38.00 per sample for one 96-well plate including bioinformatics with an addition cost of approximately US$4.50 per sample for DNA extraction using a Qiagen® DNeasy Plant Mini Kit extraction kit and an additional $0.93 per sample for labour. In relation, CTAB costs US$0.80 per genotype for DNA extraction, assessed by the Abarshi et al. (2010). CTAB is more laborious and takes longer giving it a higher labour cost per sample at US$1.25. However, PCR reagent costs were assessed by Duncan et al. (2012) at US$1.22 for a total cost of US$3.27 per sample for SRAP opposed to US$43.49 for GBS. Comparing the overall resolution between the two methods at ∼77 and 68 % similarity, respectively, SRAP appears to be as effective for separating diverse genotypes into distinct clusters for breeding purposes with a substantially lower cost. Despite the monetary difference, the similarity in clustered genotypes between each method lends credibility to both methods. Since these methods are roughly a decade apart, yet still produce similar results, we can infer that these genotypic methods are comparable when investigating heterotic pool placement based on cluster analysis and genetic distance in *B. napus* genotypes.

These current heterotic clusters as defined by SRAP and GBS may prove useful for the development of hybrid *B. napus* cultivars based on genetic distance. Future investigations need to concentrate on the accuracy of genotypic placement through inter-cluster and intra-cluster hybrids with the concurrent measure of hybrid heterosis over parental values to gauge the degree that genetic distance influences heterotic gain.

## Electronic supplementary material

Supplemental Table 1 (DOCX 26 kb)

Supplemental Table 2 (DOCX 25 kb)

Supplemental Fig. 1 Acrylamide gel featuring polymorphic DNA bands amplified using sequence related amplified polymorphism through the polymerase chain reaction with primers EM1 and BG11 visualized through autoradiography with an ABI Prism 3130XL in association with GenScan® software (V.3.7). Grey rows (6 rows) represent polymorphic bands chosen to differentiate 79 *Brassica napus* genotypes (GIF 178 kb)

High resolution image (TIFF 115 kb)

Supplemental Fig. 2 1000 bootstrap replication of the neighbour joining cluster analysis based on 293 polymorphic bands obtained through sequence related amplified polymorphism. Consensus tree construction was implemented in Geneious V.8.05 over 1000 replicates with percent threshold set to 0. Node lengths equal percent commonality over 1000 trees. Numbers and colours have been added for ease of viewing. Each genotype is either a maintainer (−B) or restorer (−R) in the *ogu*-INRA pollination control system (GIF 57 kb)

High resolution image (TIFF 49004 kb)

Supplemental Fig. 3 1000 bootstrap replication of the neighbour joining cluster analysis based on genotyping-by-sequencing 80,005 SNPs. Node lengths equal percent commonality over 1000 replicates visualized in Geneious V.8.05. Distinct clusters have been colour coded for ease of viewing. Each genotype is either a maintainer (−B) or restorer (−R) in the *ogu*-INRA pollination control system (GIF 56 kb)

High resolution image (TIFF 49153 kb)
