# Supplementary material for: Characterization of Brassica napus L. genotypes utilizing sequence-related amplified polymorphism and genotyping by sequencing in association with cluster analysis
Source: Mol Breed. 2016 Nov 10;36(11):155. doi: 10.1007/s11032-016-0576-6 (PMC5104778; doi:10.1007/s11032-016-0576-6)
Supplement: Supplementary file 1 — (DOCX 26 kb) [file 11032_2016_576_MOESM1_ESM.docx]

**Supplemental Table 1:** Forward and reverse sequence related amplified polymorphism primers for the polymerase chain reaction used in combination with each other to produce 293 polymorphic bands.

| **Forward Primers:** | |
| --- | --- |
| SA7 | 5'-CGCAAGACCCACCACAA-3' |
| EM1 | 5'-GACTGCGTACGAATTCAAT-3' |
| bg23 | 5'-ATTCAAGGAGAGTGCGTGG-3' |
| ME2 | 5'-TGAGTCCAAACCGGAGC-3' |
| ODD3 | 5'-CCAAAACCTAAAACCAGGA-3' |

| **Reverse Primers:** | |  |  |
| --- | --- | --- | --- |
| BG01 | 5'-TTTCAGGAGCAGATGGTGG-3' | BG10 | 5'-AGTTGGACATTATTGGCAGC-3' |
| BG11 | 5'-AGTTGGACATTATTGGCAGC-3' | BG32 | 5'-AGTTGGACATTATTGGCAGC-3' |
| BG33 | 5'-AGTTGGACATTATTGGCAGC-3' | BG35 | 5'-AGTTGGACATTATTGGCAGC-3' |
| BG37 | 5'-AGTTGGACATTATTGGCAGC-3' | BG38 | 5'-AGTTGGACATTATTGGCAGC-3' |
| BG39 | 5'-AGTTGGACATTATTGGCAGC-3' | BG40 | 5'-AGTTGGACATTATTGGCAGC-3' |
| BG41 | 5'-AGTTGGACATTATTGGCAGC-3' | BG45 | 5'-AGTTGGACATTATTGGCAGC-3' |
| BG60 | 5'-AGTTGGACATTATTGGCAGC-3' | BG62 | 5'-AGTTGGACATTATTGGCAGC-3' |
| BG70 | 5'-AGTTGGACATTATTGGCAGC-3' | BG72 | 5'-AGTTGGACATTATTGGCAGC-3' |
| BG4 | 5'-AGTTGGACATTATTGGCAGC-3' | BG76 | 5'-AGTTGGACATTATTGGCAGC-3' |
| BG75 | 5'-AGTTGGACATTATTGGCAGC-3' | PM018 | 5'-AAGCGATCAAAGCGGGTG-3' |
| PM029 | 5'-GATGAGAAACTGAACGAGG-3' | PM032 | 5'-AAGTATGGGGTTGGGTTTC-3' |
| PM034 | 5'-GGGACGAACAGACAACGA-3' | PM117 | 5'-GTCAGCAAGTTTCACGGTT-3' |
