# Supplementary material for: Characterization of Brassica napus L. genotypes utilizing sequence-related amplified polymorphism and genotyping by sequencing in association with cluster analysis
Source: Mol Breed. 2016 Nov 10;36(11):155. doi: 10.1007/s11032-016-0576-6 (PMC5104778; doi:10.1007/s11032-016-0576-6)
Supplement: Supplementary file 2 — (DOCX 25 kb) [file 11032_2016_576_MOESM2_ESM.docx]

**Supplemental Table 2:** Forward and reverse primer combinations used for sequence related amplified polymorphism to generate 293 polymorphic bands amplified by PCR with 79 *Brassica napus* genotypes.

| **Forward Primer** | | | | |
| --- | --- | --- | --- | --- |
| EM1 | bg23 | Sa7 | ME2 | ODD3 |
| **Reverse Primer** | | | | |
| BG10 | BG33 | BG1 | BG11 | PM32 |
| BG11 | BG4 | BG60 | BG62 | PM34 |
| BG33 | BG41 | BG35 | BG70 |  |
| BG45 | BG62 | BG39 |  |  |
| BG72 | BG37 | BG40 |  |  |
| BG75 | BG38 | BG41 |  |  |
| BG76 | PM18 | PM18 |  |  |
| BG32 | PM117 | PM29 |  |  |
