# Supplementary figures and images for: Characterization of Brassica napus L. genotypes utilizing sequence-related amplified polymorphism and genotyping by sequencing in association with cluster analysis
Source: Mol Breed. 2016 Nov 10;36(11):155. doi: 10.1007/s11032-016-0576-6 (PMC5104778; doi:10.1007/s11032-016-0576-6)

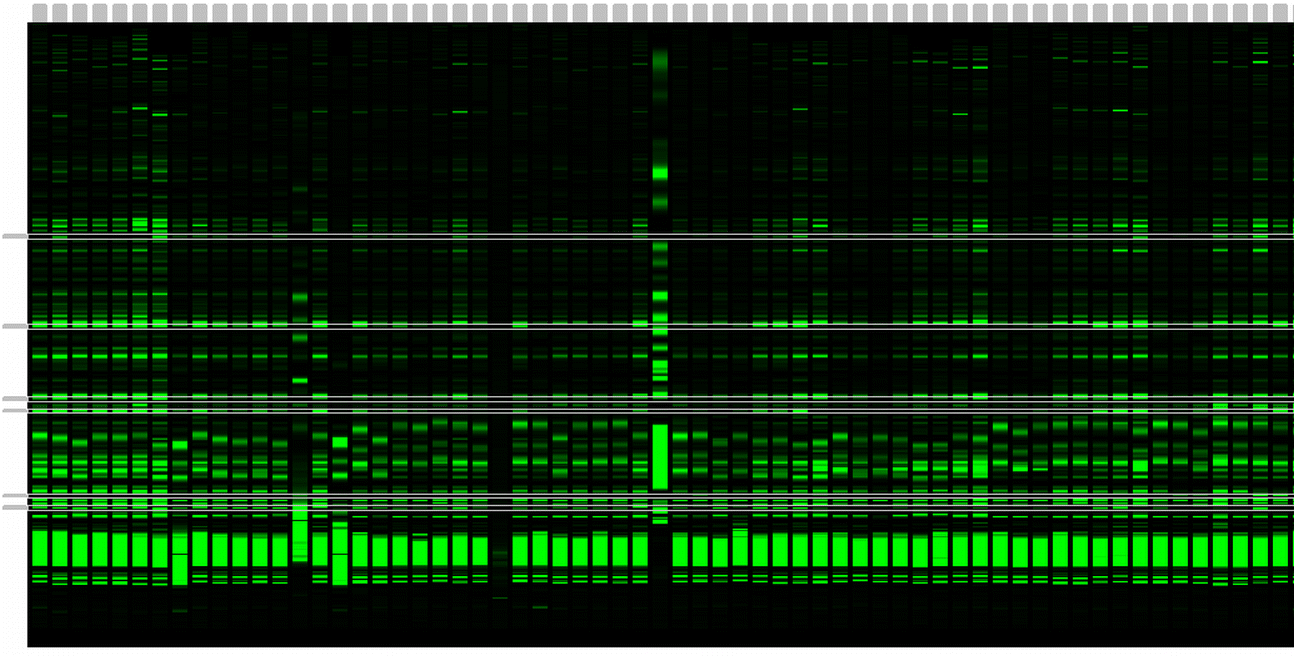

Supplement: Supplementary file 3 — Acrylamide gel featuring polymorphic DNA bands amplified using sequence related amplified polymorphism through the polymerase chain reaction with primers EM1 and BG11 visualized through autoradiography with an ABI Prism 3130XL in association with GenScan® software (V.3.7). Grey rows (6 rows) represent polymorphic bands chosen to differentiate 79 Brassica napus genotypes (GIF 178 kb) [file 11032_2016_576_Fig3_ESM.gif]

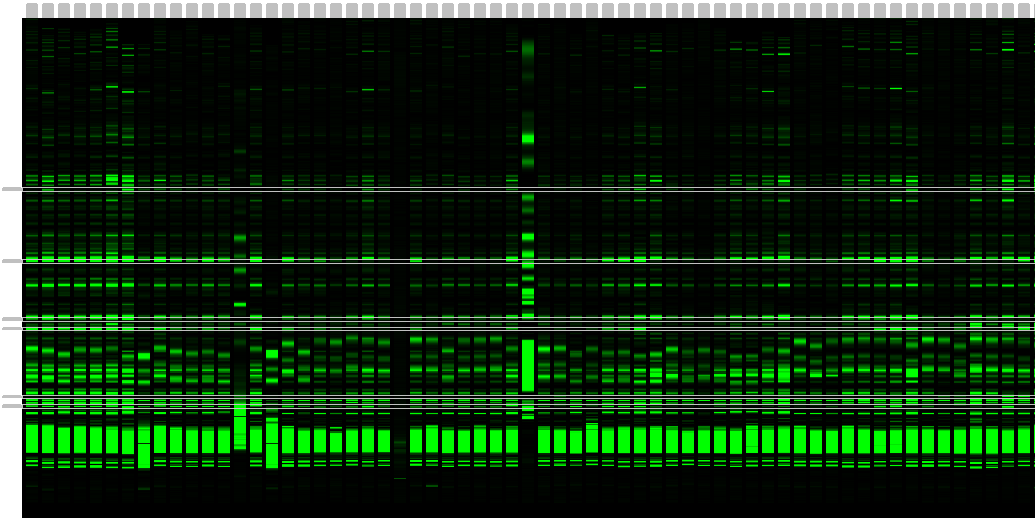

Supplement: Supplementary file 4 — High resolution image (TIFF 115 kb) [file 11032_2016_576_MOESM3_ESM.tif]

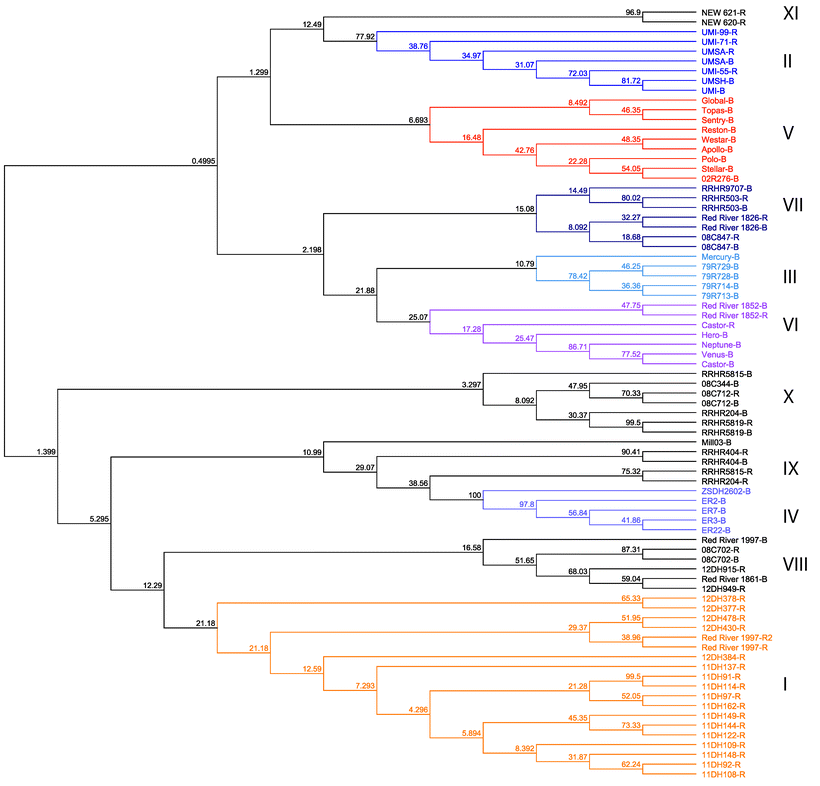

Supplement: Supplementary file 5 — 1000 bootstrap replication of the neighbour joining cluster analysis based on 293 polymorphic bands obtained through sequence related amplified polymorphism. Consensus tree construction was implemented in Geneious V.8.05 over 1000 replicates with percent threshold set to 0. Node lengths equal percent commonality over 1000 trees. Numbers and colours have been added for ease of viewing. Each genotype is either a maintainer (−B) or restorer (−R) in the ogu-INRA pollination control system (GIF 57 kb) [file 11032_2016_576_Fig4_ESM.gif]

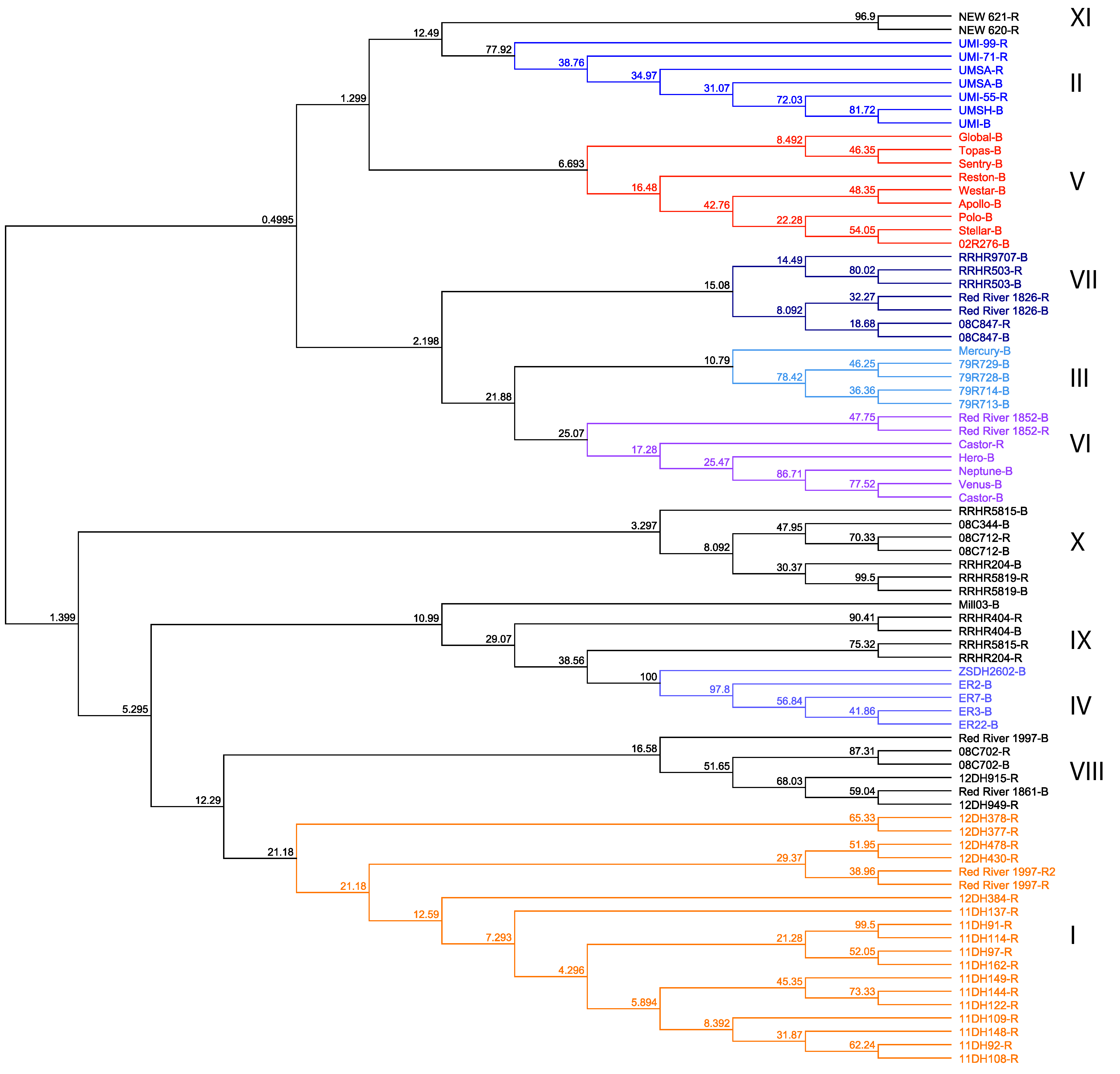

Supplement: Supplementary file 6 — High resolution image (TIFF 49004 kb) [file 11032_2016_576_MOESM4_ESM.tif]

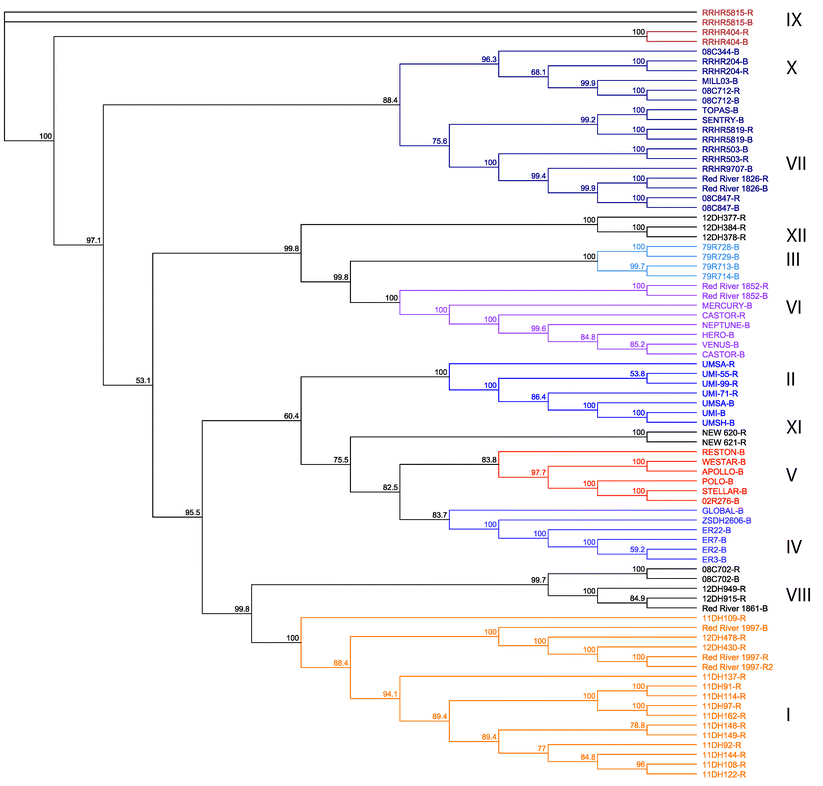

Supplement: Supplementary file 7 — 1000 bootstrap replication of the neighbour joining cluster analysis based on genotyping-by-sequencing 80,005 SNPs. Node lengths equal percent commonality over 1000 replicates visualized in Geneious V.8.05. Distinct clusters have been colour coded for ease of viewing. Each genotype is either a maintainer (−B) or restorer (−R) in the ogu-INRA pollination control system (GIF 56 kb) [file 11032_2016_576_Fig5_ESM.gif]

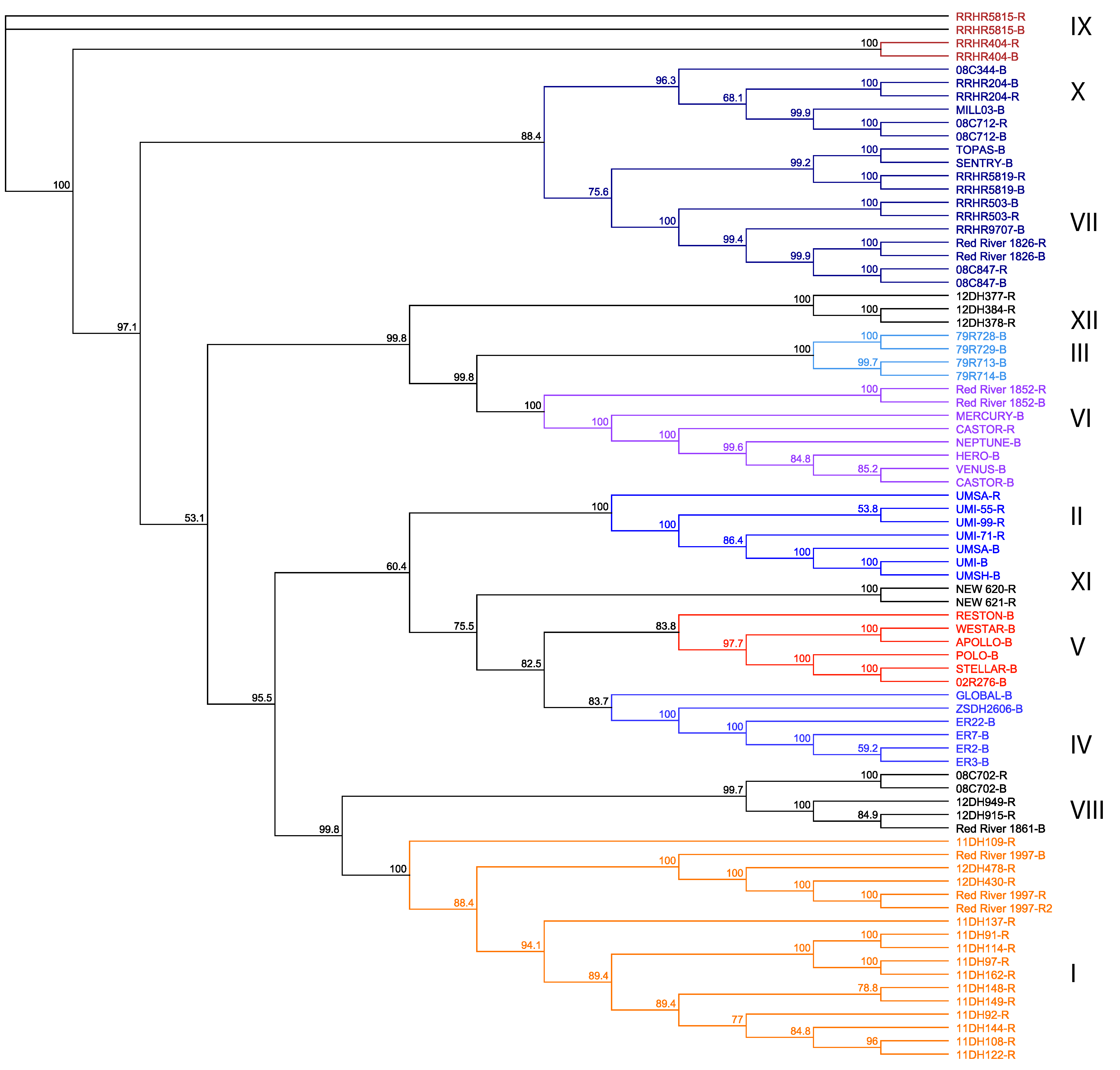

Supplement: Supplementary file 8 — High resolution image (TIFF 49153 kb) [file 11032_2016_576_MOESM5_ESM.tif]
